# Imatinib and Dasatinib Provoke Mitochondrial Dysfunction Leading to Oxidative Stress in C2C12 Myotubes and Human RD Cells

**DOI:** 10.3389/fphar.2020.01106

**Published:** 2020-07-23

**Authors:** Jamal Bouitbir, Miljenko Valentin Panajatovic, Theo Frechard, Noëmi Johanna Roos, Stephan Krähenbühl

**Affiliations:** ^1^ Division of Clinical Pharmacology & Toxicology, University Hospital of Basel, Basel, Switzerland; ^2^ Department of Biomedicine, University of Basel, Basel, Switzerland; ^3^ Swiss Centre for Applied Human Toxicology (SCAHT), University of Basel, Basel, Switzerland

**Keywords:** imatinib, dasatinib, myotoxicity, electron transport chain (ETC), reactive oxygen species (ROS), apoptosis, atrophy

## Abstract

Tyrosine kinase inhibitors (TKIs) can cause skeletal muscle toxicity in patients, but the underlying mechanisms are mostly unclear. The goal of the current study was to better characterize the role of mitochondria in TKI-associated myotoxicity. We exposed C2C12 murine myoblasts and myotubes as well as human rhabdomyosarcoma cells (RD cells) for 24 h to imatinib (1–100 µM), erlotinib (1–20 µM), and dasatinib (0.001–100 µM). In C2C12 myoblasts, imatinib was membrane toxic at 50 µM and depleted the cellular ATP pool at 20 µM. In C2C12 myotubes exposed to imatinib, ATP depletion started at 50 µM whereas membrane toxicity was not detectable. In myoblasts and myotubes exposed to dasatinib, membrane toxicity started at 0.5 µM and 2 µM, respectively, and the ATP drop was visible at 0.1 µM and 0.2 µM, respectively. When RD cells were exposed to imatinib, ATP depletion started at 20 µM whereas membrane toxicity was not detectable. Dasatinib was membrane toxic at 20 µM and depleted the cellular ATP pool already at 0.5 µM. Erlotinib was not toxic in both cell models. Imatinib (20 µM) and dasatinib (1 µM) reduced complex I activity in both cell models. Moreover, the mitochondrial membrane potential (*Δψ*m) was dissipated for both TKIs in myotubes. In RD cells, the *Δψ*m was reduced only by dasatinib. Both TKIs increased mitochondrial superoxide accumulation and decreased the mitochondrial copy number in both cell lines. In consequence, they increased protein expression of superoxide dismutase (SOD) 2 and thioredoxin 2 and cleavage of caspase 3, indicating apoptosis in C2C12 myotubes. Moreover, in both cell models, the mRNA expression of *Sod1* and *Sod2* increased when RD cells were exposed to dasatinib. Furthermore, dasatinib increased the mRNA expression of *atrogin-1* and *murf-1*, which are important transcription factors involved in muscle atrophy. The mRNA expression of *atrogin-1* increased also in RD cells exposed to imatinib. In conclusion, imatinib and dasatinib are mitochondrial toxicants in mouse C2C12 myotubes and human RD cells. Mitochondrial superoxide accumulation induced by these two TKIs is due to the inhibition of complex I and is probably related to impaired mitochondrial and myocyte proliferation.

## Introduction 

Tyrosine kinases (TKs) are a large family of enzymes, which are responsible for catalyzing phosphorylation reactions of the tyrosine molecules using ATP (Robinson et al., 2000). Through this phosphorylation, they play key roles in cell proliferation, differentiation, migration, metabolism, and programmed cell death (Hubbard and Miller, 2007). This enzyme family has become one of the most important drug targets in the past 20 years because mutations, overexpression, and dysregulation of TKs play crucial roles in the pathogenesis of cancer (Blume-Jensen and Hunter, 2001; Roskoski, 2020). The development of targeted cancer treatments with low molecular weight tyrosine kinase inhibitors (TKIs) allows impairing cancer cell proliferation and tumor progression in a specific fashion. Since cancer is one of the leading causes of death in the world, the development of TKIs has provided a large step forward in cancer treatment with significant survival benefit (Andrews and Lipton, 2019; Dagenais et al., 2020). To date, there are approximately four dozens FDA-approved TKIs (Roskoski, 2020). As the first approved TKI of this new generation of anticancer drugs in 2001, imatinib is used for the treatment of Philadelphia-chromosome-positive chronic myelogenous leukemia (CML), which then opened the way for the development of many more TKIs (Kantarjian et al., 2002). Later, erlotinib and dasatinib, both multi-targeted TKIs, were approved in the USA and in Europe for the treatment of patients with lung cancer and CML, respectively (Duckett and Cameron, 2010).

While TKIs are better tolerated than the conventional cytotoxic treatments, they also cause several adverse reactions including gastrointestinal, cardiovascular, dermatologic, and hepatic toxicities that patients may not tolerate (Caldemeyer et al., 2016). TKIs are also recognized to induce skeletal muscle toxicity in patients (Moscetti et al., 2011; AlJohani et al., 2015; Pasnoor et al., 2018). For instance, it has been reported that myalgia occurs in 32% and creatine kinase elevations in 45% of patients treated with imatinib (Adenis et al., 2012). Specifically, skeletal muscle disorders are observed in up to 80% of CML patients treated with TKIs (Kekale et al., 2015; Janssen et al., 2019). The symptoms of TKI-associated muscle disturbances, which include mainly fatigue and weakness, can contribute to both impaired quality of life and diminished treatment adherence (Efficace et al., 2013; Kekale et al., 2015). Although the mechanisms responsible for the TKIs-associated myotoxicity are currently not definitively clarified, mitochondrial dysfunction has been proposed to play a key role in the imatinib-induced skeletal muscle toxicity. Indeed, it has been shown that imatinib decreased the complex IV activity of the electron transport chain (ETC) in mouse C2C12 myotubes, indicating that mitochondria may be responsible for skeletal muscle toxicity (Damaraju et al., 2018).

Because mechanistic toxicological studies concerning erlotinib and dasatinib, which are recognized to cause skeletal muscle disturbances in patients, are missing, our goal was to investigate the skeletal muscle toxicity of these drugs. We performed these investigations in comparison to imatinib using C2C12 murine myoblasts and myotubes as well as human rhabdomyosarcoma (RD) cells. C2C12 myoblasts are a well-established model to investigate skeletal muscle toxicity (Schirris et al., 2015; Damaraju et al., 2018). Upon serum withdrawal, C2C12 myoblasts differentiate into polynucleated myotubes, which contain sarcomeres and are able to contract and to generate force (Sanvee et al., 2019). Therefore, we aimed to find out mechanisms responsible for the TKIs-associated myotoxicity with a special attention to mitochondrial toxicity and atrophy. For that, we determined the mitochondrial membrane potential, the activity of the electron transport chain, mitochondrial superoxide accumulation, and markers of apoptosis and atrophy in differentiated C2C12 myotubes and human RD cells.

## Materials and Methods

### Chemicals

Imatinib mesylate, erlotinib mesylate, and dasatinib were acquired from Sequoia research products (Pangbourne, UK). Stock solutions of these drugs were prepared in dimethylsulfoxide (DMSO) and were stored at −20°C. All other chemicals were supplied by Sigma-Aldrich (Buchs, Switzerland), except where indicated.

### Culturing of Mouse C2C12 Cells

C2C12 murine myoblasts (American Type Culture Collection [ATCC], USA) were grown in confluency in growth medium containing Dulbecco’s Modified Eagle Medium (DMEM, Gibco, UK), GlutaMAX, 10% fetal bovine serum (FBS), and 1% HEPES (Gibco, UK). Cells were incubated at 37°C in a humidified air with 5% CO_2_. The cell number was determined using a Neubauer hemocytometer and viability was checked using the trypan blue exclusion method. Three days after seeding, the medium was replaced by differentiation medium (DM) containing DMEM-Glutamax, 1% HEPES, 2% horse serum (Gibco, UK), and 0.03% insulin (stock: 10 mg/ml) in order to obtain myotubes. Three days later, we removed the medium and added the differentiation medium without insulin (Sanvee et al., 2019). Myotubes were then treated for 24 h with TKIs in serum-free differentiation medium.

### Culturing of Human RD Cells

Human rhabdomyosarcoma cells (RD cells) were acquired from ATCC (Manassas, USA). The RD cells were kept in growth medium DMEM and 10% FBS. Cells were incubated at 37°C in a humidified air with 5% CO_2_. Cells were passaged using trypsin upon reaching approximately 80% confluence. We measured the cell number using a Neubauer hemocytometer and we then checked viability using the trypan blue exclusion method.

### Membrane Toxicity

We evaluated the membrane toxicity using the Toxilight assay from Lonza (Basel, Switzerland) according to the manufacturer’s protocol. This method measures the release of adenylate kinase (AK) in the medium, which indicates the integrity of the plasma membrane. We used 0.1% Triton X as a positive control. After 24 h exposure to the TKIs, we transferred 20 μl of the supernatant to a new opaque 96-well plate. Then, we added 50 μl of assay buffer to each well. After incubation for 5 min, we measured the luminescence using a Tecan M200 Pro Infinity plate reader (Männedorf, Switzerland). All data were normalized to 0.1% Triton X (set at 100% cell lysis).

### Intracellular ATP Content

We evaluated the intracellular ATP content using the CellTiterGlo Luminescent cell viability assay (Promega, Switzerland), in accordance with the manufacturer’s instructions. Briefly, after 24 h exposure to the TKIs, 80 μl of assay buffer was added to each 96-well containing 80 μl culture medium. After incubation in the dark for 15 min, we measured the luminescence using a Tecan M200 Pro Infinity plate reader (Männedorf, Switzerland). All data were normalized to the negative control condition containing 0.1% DMSO.

### Mitochondrial Membrane Potential

The mitochondrial membrane potential (*Δψ*m) was measured using an O2k-Fluorescence LED2 module equipped with Fluorescence-Sensor Blue (Oroboros instruments, Innsbruck, Austria). In order to access *Δψ*m, we used safranin as a fluorescent probe that accumulates in energized mitochondria according to the inside negative potential (Perevoshchikova et al., 2009). The Fluorescence-Sensor Blue is equipped with a filter set for safranin (excitation and emission wavelengths of 495 nm and 587 nm, respectively). We performed the experiments in MiR05 buffer as a mitochondrial respiration medium. MiR05 buffer contains 0.5 mM EGTA, 3 mM magnesium chloride, 20 mM taurine, 10 mM potassium dihydrogen phosphate, 20 mM HEPES, 110 mM sucrose, 1 g/I fatty-acid free bovine serum albumin, and 60 mM lactobionic acid at pH 7.1 (Pesta and Gnaiger, 2012). For the calibration, a 200 μM stock solution of safranin dissolved in distilled water was added in each chamber and titrated in five steps into the O2k chamber, up to a final concentration of safranin at 2 μM. During the calibration step, we observed a linear increase of the fluorescence, reflecting the concentration of safranin in the chamber. After the calibration, we added C2C12 myotubes and human RD cells permeabilized with digitonin. As a first step, we added glutamate (10 mM) and malate (2 mM) as substrates for complex I of the electron transport chain (ETC). The fluorescence signal showed a sharp decrease, reflecting an increase of *Δψ*m. At this point, we stimulated the respiratory chain by adding adenosine-diphosphate (ADP) at 2.5 mM. This was associated with a decrease of *Δψ*m (increase of fluorescence) since protons are shuttled through the ATP synthase to drive ATP production. Data show the *Δψ*m under glutamate/malate and ADP as substrates. Results were then normalized to the negative control condition containing 0.1% DMSO.

### Activity of Specific Enzyme Complexes of the Mitochondrial Electron Transport Chain

An Oxygraph-2k high-resolution respirometer equipped with DataLab software was used to analyze the activity of specific enzyme complexes of the respiratory chain (Oroboros instruments, Innsbruck, Austria). After 24 h of treatment, C2C12 myotubes and human RD cells were placed in a thermostated oxygraphic chamber containing MiR05 buffer at 37°C with continuous stirring, as described previously (Pesta and Gnaiger, 2012). After the permeabilization with digitonin, we determined the activity of complexes I, II, III, and IV. First, we assessed the activity of complexes I and III using L-glutamate and malate (10 mM and 2 mM, respectively) as substrates. We added then ADP (2.5 mM) and rotenone (0.5 μM) as an inhibitor of complex I. Then, we added duroquinol (500 mM) as an artificial substrate of complex III followed by antimycin A (2.5 μM) as an inhibitor of complex III. In a second run, we assessed the activity of complexes II and IV using succinate (10 mM) in the presence of rotenone (0.5 μM) as substrates followed by the addition of ADP (2.5 mM). We added then malonate (5 mM) as an inhibitor of complex II. Later, we added N,N,N′,N′-tetramethy-p-phenylenediamine dihydrochloride (TMPD)/ascorbate (0.5 mM and 2 mM, respectively) to investigate complex IV, which was then inhibited by potassium cyanide (1 mM). The integrity of the outer mitochondrial membrane was investigated by assessing the effect of cytochrome c (10 μM) on mitochondrial respiration. Protein concentrations were determined using the Pierce BCA Protein Assay kit (Thermo Fisher Scientific, Basel, Switzerland). We expressed the respiratory rates in pmol O_2_ × s^−1^ × mg^−1^ protein.

### Mitochondrial Superoxide Accumulation

The MitoSOX™ Red fluorophore dye (Thermo Fisher Scientific, Basel, Switzerland) was used for the determination of mitochondrial superoxide accumulation. Upon treatment with TKIs for 24 h, the medium was removed and the cells were rinsed with PBS (Phosphate buffer solution). As a positive control, we exposed C2C12 myotubes and RD cells to 100 µM antimycin for 30 min, which is a well-known inhibitor of complex III of the ETC (Bouitbir et al., 2016). Next, MitoSOX reagent (2.5 μM) prepared in PBS was added to each well and we incubated the plate for 10 min at 37°C with light protection. We measured the fluorescence using a Tecan M200 Infinite Pro plate reader (Tecan, Männedorf, Switzerland) at excitation and emission wavelengths of 510 nm and 580 nm, respectively. The results were normalized to the protein content, which was quantified by the Pierce BCA Protein Assay kit (Thermo Fisher Scientific, Basel, Switzerland) and then to DMSO 0.1% treated control cells.

### Mitochondrial DNA Copy Number

We determined mitochondrial DNA copy number using quantitative polymerase chain reaction (qPCR), as described previously (Quiros et al., 2017). Briefly, we extracted total DNA using the DNeasy Blood and Tissue Kit (Qiagen, Hombrechtikon, Switzerland), in accordance with the manufacturer’s instructions. We measured the DNA concentration in the samples spectrophotometrically at 260 nm with the NanoDrop 2000 (Thermo Scientific, Wohlen, Switzerland). We diluted the samples to a final concentration of 10 ng/μl DNA. The DNA was then subjected to qPCR in triplicate (see **Table 1** for primers) using SYBR Green (Roche Diagnostics, Rotkreuz, Basel) and performed with the ViiA ™ 7 Real-Time PCR System (Applied Biosystems, Waltham, MA, USA). Quantification of mitochondrial copy number was performed as described previously (Quiros et al., 2017).

**Table 1:** Sequences of primers used for the quantitative RT-PCR and qPCR.

Target gene	Forward primer 5′ ———>3′ Reverse primer 5′ ———>3′	Species
*18s*	TTGCTGACAGGATGCAGAAG CAGTGAGGCCAGGATAGAGC	mouse
*Pgc-1α*	AAT GCA GCG GTC TTA GCA CT ACG TCT TTG TGG CTT TTG CT	mouse
*Pgc-1β*	TGC GGA GAC ACA GAT GAA GA GGC TTG TAT GGA GGT GTG GT	mouse
*Nrf1*	TTA CTC TGC TGT GGC TGA TGG CCT CTG ATG CTT GCG TCG TCT	mouse
*Nrf2*	CGA GAT ATA CGC AGG AGA GGT GCT CGA CAA TGT TCT CCA GCT T	mouse
*Tfam*	GCT GAT GGG TAT GGA GAA G GAG CCG AAT CAT CCT TTG C	mouse
*Atrogin-1*	AGTGAGGACCGGCTACTGTG GATCAAACGTTGCGAATCT	mouse
*Murf-1*	CCTGCAGAGTGACCAAGGA GGCGTAGAGGGTGTCAAACT	mouse
ND1	GCAGCTTAACATTCCGCCCAATCA TACTGGTTGGCCTCCGATTCATGT	mouse
Hexokinase	TGTGGGTGATCTGGTGATTGTGGT AGGCATTTCAGGATACGCTCAGCA	mouse
*β actin*	GATCATTGCTCCTCCTGAGC ACTCCTGCTTGCTGATCCAC	human
*Pgc-1α*	AGGCTAGTCCTTCCTCCATGCGTT GGCTGGTGCCAGTAAGAG	human
*Pgc-1β*	GAGTCAAAGTCGCTGGCATC AACTATCTCGCTGACACGCA	human
*Nrf1*	AAACGCAAACACAGGCCACAGCC TCAGCCAATGTGGCTACTGTTGCCC	human
*Nrf2*	TGAGCCCAGTATCAGCA CA AGTGAAATGCCGGAGTCAG	human
*Tfam*	TTGCCCAGCGTTGGAGGGAAC CCTGCCACTCCGCCCTATAAGC	human
*Atrogin-1*	GACTTCTCAACTGCCATTC TCGTCTCCATCCGATACAC	human
*Murf-1*	GCTGAGCCAGAAGTTTGA CAGGGCGTCTGCTATGTG	human
*Sod1*	TGTTGGAGACTTGGGCAATG CAATGATGCAATGGTCTCCTGA	human
*Sod2*	TTTAGTCCCTGGTGTTCCCC CTTCACCGAAAACTCCAGGC	human
*Trx1*	GTTGACTTCTCAGCCACGTG TCACCCACCTTTTGTCCCTT	human
*Trx2*	CCCACACTGAAATCCCCTCT CCCCTGCTCAGAAAACCAAC	human
Cox1	TTCGCCGACCGTTGACTATT AAGATTATTACAAATGCATGGGC	human
ASPOLG	GAGCTGTTGACGGAAAGGAG CAGAAGAGAATCCCGGCTAAG	human

### Quantitative Real Time PCR

Total RNA was obtained from C2C12 myotubes and human RD cells using the Qiagen RNeasy mini extraction kit according to the manufacturer’s instructions (Qiagen, Hombrechtikon, Switzerland). We measured the quantity and purity of RNA with the NanoDrop 2000 (Thermo Scientific, Wohlen, Switzerland). For the reverse transcription, cDNA was synthesized from 1 μg of total RNA using the Omniscript RT kit (QiagenGmbH, Hilden, Germany). Next, cDNA was mixed with forward and reverse primers (0.3 μM), SYBR Green (Roche Diagnostics, Rotkreuz, Switzerland) as a fluorescent dye for the measurement of duplex DNA formation. The real-time PCR measurement was performed in triplicate with the ViiA ™ 7 Real-Time PCR System (Applied Biosystems, Waltham, MA, USA). Primer sequences were designed using information contained in the public database in the GeneBank of the National Center for Biotechnology Information (NCBI). The sequences of primer sets used are listed in **Table 1**. Quantification of relative gene expression levels was performed using the ΔΔCt method with *18s* gene and *β actin* as housekeeping genes in C2C12 myotubes and in RD cells, respectively (Ramakers et al., 2003).

### Western Blotting

Upon treatment with TKIs, C2C12 myotubes were lysed using radioimmunoprecipitation assay (RIPA: 150 mM sodium chloride, 1.0% NP-40, 0.5% sodium deoxycholate, 0.1% sodium dodecyl sulphate, 50 mM Tris; pH 8.0) buffer on ice for 15 min and then centrifuged to obtain protein samples. Following the collection of supernatants, BCA Pierce assay was used to quantify the protein concentration of each sample. 10 µg of proteins was loaded onto the commercially available 4–12% NuPAGE Bis-Tris gels (Invitrogen, Basel, Switzerland). The gel was run at 140 V and after the separation, the gel was electroblotted to a nitrocellulose membrane using the Trans-Blot Turbo Blotting System (Bio-Rad, Cressier, Switzerland). Proteins were then immunodetected using antibodies against superoxide dismutase 1 (SOD1) (ab51254, abcam, 1:5,000), SOD2 (#13194, cell signaling, 1:2,000), thioredoxin 1 (TRX1) (ab109385, abcam, 1:10,000), TRX2 (ab185544, abcam, 1:10’000), full and cleaved caspase 3 (#9665S, cell signaling, 1:500), and GAPDH (sc-365062, Santa Cruz biotechnology, 1:1,000). Membranes were then probed with secondary HRP-conjugated antibodies (Santa Cruz Biotechnologies, USA) for 1 h (1:2,000 in the blocking solution). We then washed the membranes and the ClarityTM Western ECL Substrate (Bio-Rad Laboratories, USA) was added to visualize the bands. Protein expression was quantified using the Fusion Pulse TS device (Vilber Lourmat, Oberschwaben, Germany).

### Statistical Analysis

Data are expressed as mean ± SEM. Statistical analysis was completed using the GraphPad Prism 8 program (GraphPad Software, San Diego, CA, USA). The results were evaluated with one-way ANOVA, followed by the comparison between incubations containing TKIs and the control group using Dunnett’s post-test procedure. P-values < 0.05 (*) were considered significant.

## Results

### Membrane Toxicity and Intracellular ATP Content in C2C12 Myoblasts and Myotubes

**Figure 1:**
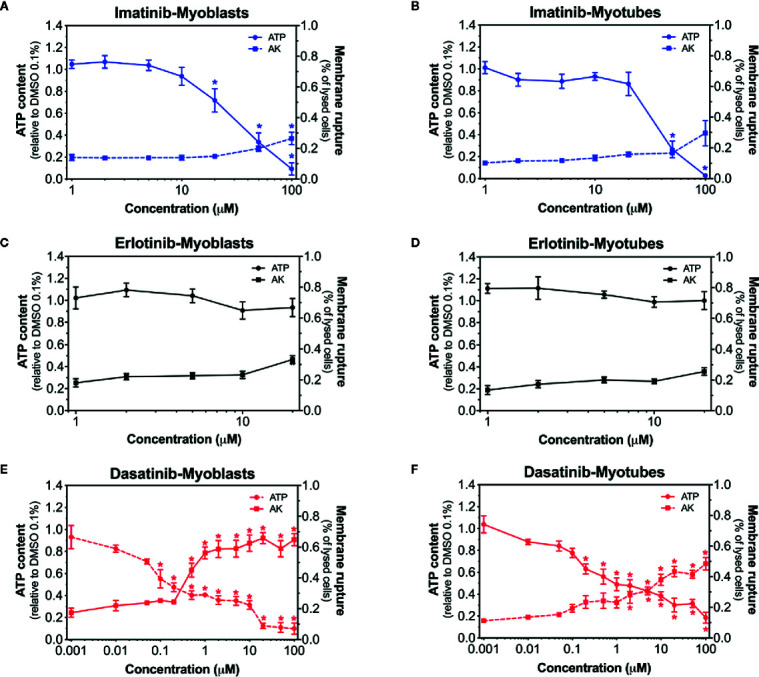
Membrane toxicity and intracellular ATP content in C2C12 myoblasts and myotubes. Membrane toxicity (AK release) and ATP content after exposure with increasing concentrations of imatinib **(A, B)**, erlotinib **(C, D),** and dasatinib **(E, F)** for 24 h in C2C12 myoblasts and myotubes, respectively. Data are expressed as % AK release in the presence of 0.1% Triton X (set at 100%) or as % ATP content in the presence of 0.1% DMSO (set at 100%). Data represent the mean ± SEM of at least four independent experiments. Treatments with TKIs were compared to 0.1% DMSO control with one-way ANOVA followed by the comparison between incubations containing TKIs and the control group using Dunnett’s post-test procedure. *p < 0.05 *versus* 0.1% DMSO control.

To elucidate the toxic effects of the three investigated TKIs, we first assessed the plasma membrane integrity and the intracellular ATP content in myoblasts and myotubes exposed to different concentrations (Bouitbir et al., 2019). After exposure of C2C12 myoblasts and myotubes for 24 h, imatinib was slightly membrane toxic and depleted the ATP content in a concentration-dependent fashion (**Figures 1A, B**). The membrane toxicity started to increase at 100 µM in myoblasts, but not in myotubes (**Figures 1A, B**). Furthermore, imatinib depleted the cellular ATP pool starting already at 20 µM in myoblasts and at 50 µM in myotubes (**Figures 1A, B**). As shown in **Figures 1C, D**, the exposure to erlotinib (investigated up to 20 µM) was only slightly toxic in myoblasts and myotubes at the highest concentration without reaching statistical significance and did not affect the cellular ATP content. Dasatinib depleted the ATP pool in a concentration-dependent manner in both myoblasts (starting at 0.1 µM) and myotubes (starting at 0.2 µM) (**Figures 1E, F**). Moreover, dasatinib was membrane toxic in myoblasts (starting at 0.5 µM) and myotubes (starting at 2 µM) (**Figures 1E, F**). These data indicated that myotubes seem to be more resistant to dasatinib and imatinib than myoblasts (**Supplementary Table 1**). Moreover, dasatinib and imatinib showed a more pronounced toxicity regarding the decrease in the intracellular ATP content when compared to membrane toxicity, a pattern suggesting mitochondrial toxicity for both TKIs (**Supplementary Table 1**).

### Membrane Toxicity and Intracellular ATP Content in Human RD Cells

**Figure 2:**
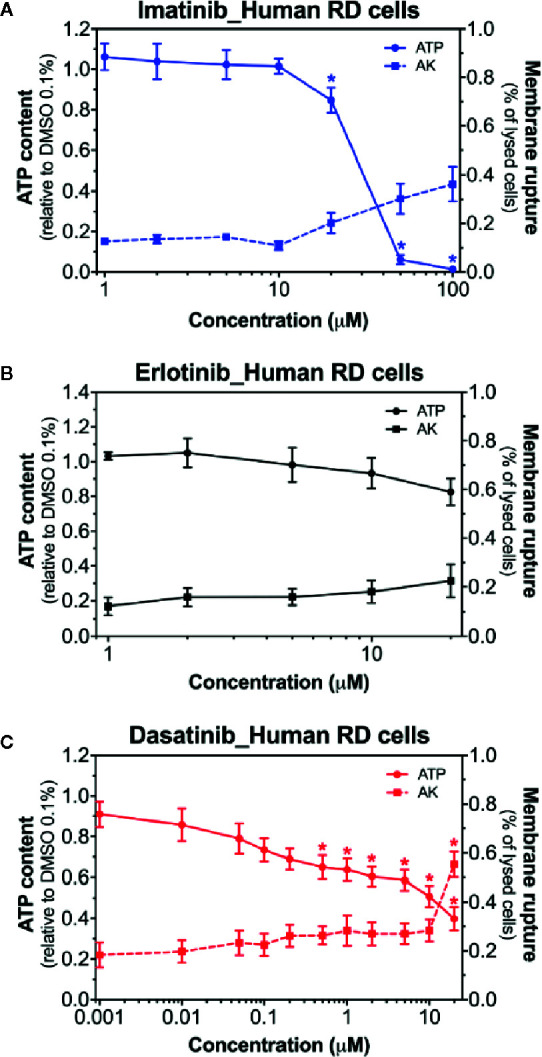
Membrane toxicity and intracellular ATP content in human RD cells. Membrane toxicity (AK release) and ATP content after exposure with increasing concentrations of imatinib **(A)**, erlotinib **(B),** and dasatinib **(C)** for 24 h in RD cells. Data are expressed as % AK release in the presence of 0.1% Triton X (set at 100%) or as % ATP content in the presence of 0.1% DMSO (set at 100%). Data represent the mean ± SEM of at least four independent experiments. Treatments with TKIs were compared to 0.1% DMSO control with one-way ANOVA followed by Dunnett’s post-test. *p < 0.05 *versus* 0.1% DMSO control.

Next, we examined the membrane toxicity and the ATP content in human RD cells (**Figure 2**). Imatinib decreased the intracellular ATP content, but exhibited only slight membrane toxicity (**Figure 2A**). When imatinib was present, almost no ATP was left at 50 µM, while only 30% of the cells were lysed at this concentration (**Figure 2A**). As observed in C2C12 myoblasts and myotubes, the exposure to erlotinib for 24 h was not membrane toxic in RD cells, even at the highest concentration used (20 µM), and also it did not affect the cellular ATP content (**Figure 2B**). The intracellular ATP pool was decreased in the presence of dasatinib in a concentration-dependent manner starting at 0.5 µM, whereas membrane toxicity was observed only at 100 µM (**Figure 2C**). Since membrane toxicity was observed at higher concentrations than ATP depletion for imatinib and dasatinib, these results suggested that mitochondrial toxicity preceded membrane toxicity (**Supplementary Table 2**). These findings in human RD cells confirmed our observations in C2C12 myoblasts and myotubes. In these cell lines, erlotinib was not toxic up to 20 µM (the highest possible concentration due to limited solubility in an aqueous milieu). Accordingly, we decided to continue our observations in C2C12 myotubes and human RD cells exposed to imatinib and dasatinib.

### Mitochondrial Membrane Potential in C2C12 Myotubes and in Human RD Cells

**Figure 3:**
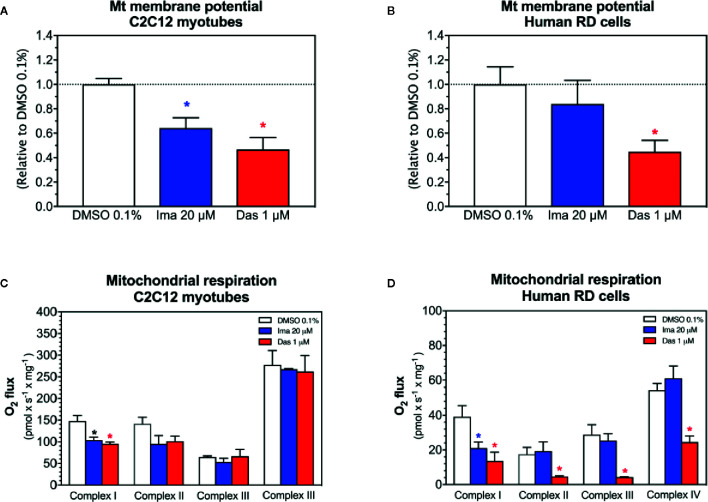
Mitochondrial membrane potential and activity of enzyme complexes of the electron transport chain in C2C12 myotubes and human RD cells exposed to imatinib and dasatinib for 24 h. **(A, B)** Mitochondrial membrane potential in C2C12 myotubes and RD cells, respectively. **(C, D)** Activity of the enzyme complexes of the electron transport chain in permeabilized myotubes and RD cells, respectively. Data represent the mean ± SEM of at least three independent experiments. Treatments with TKIs were compared to 0.1% DMSO control with one-way ANOVA followed by Dunnett’s post-test. *p < 0.05 *versus* 0.1% DMSO control. Ima, imatinib; Das, dasatinib.

As a next step, as an assessment of the mitochondrial membrane potential, the effect of imatinib and dasatinib on safranin accumulation by mitochondria was determined in C2C12 myotubes and in human RD cells (Perevoshchikova et al., 2009; Krumschnabel et al., 2014). We found that the exposure of myotubes to dasatinib (1 µM) for 24 h showed decreased *ΔΨ*_m_ in C2C12 myotubes (**Figure 3A**) and in RD cells (**Figure 3B**). Imatinib (20 µM) decreased *ΔΨ*_m_ in C2C12 myotubes (**Figure 3A**), but not in RD cells (**Figure 3B**).

### Activity of Enzyme Complexes of the ETC in C2C12 Myotubes and in Human RD Cells

We observed decreased cellular ATP content and dissipation of the mitochondrial membrane potential, which could be induced by impaired mitochondrial respiratory chain (Haegler et al., 2017). Therefore, the respiratory capacities through the complexes of the ETC were measured in C2C12 myotubes and human RD cells using a high-resolution respirometry system. Myotubes and RD cells were exposed to imatinib (20 µM) and dasatinib (1 µM) for 24 h. In C2C12 myotubes, both TKIs significantly impaired the activity of complex I of the ETC (**Figure 3C**). Moreover, the complex II activity of the ETC was also decreased for both TKIs, but without reaching statistical significance (**Figure 3C**). However, complexes III and IV of the ETC were not affected in C2C12 myotubes exposed to imatinib and dasatinib (**Figure 3C**). In RD cells, both TKIs significantly impaired the activity of complex I of the ETC (**Figure 3D**). Moreover, dasatinib decreased the activity of complexes II, III, and IV (**Figure 3D**). These findings confirmed the mitochondrial toxicity for both TKIs in both cell models.

### Mitochondrial Superoxide Accumulation in C2C12 Myotubes and in Human RD Cells

**Figure 4:**
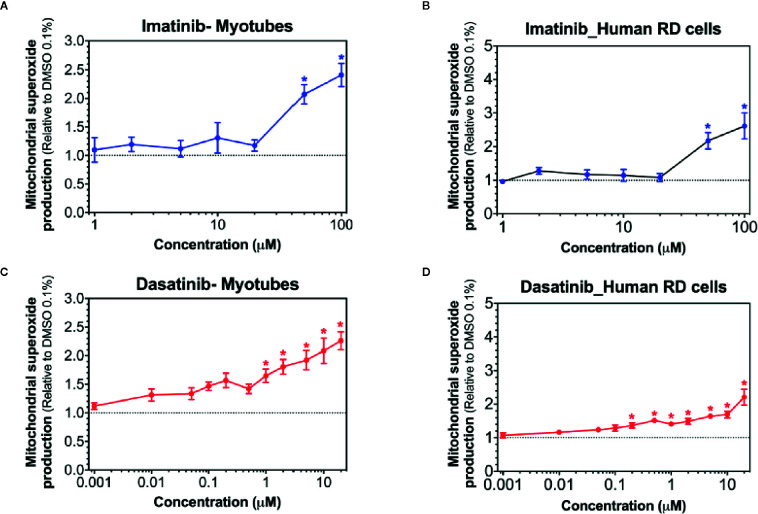
Mitochondrial superoxide accumulation in C2C12 myotubes and human RD cells. Mitochondrial superoxide accumulation during exposure to increased concentrations of imatinib **(A, B)**, and dasatinib **(C, D)** for 24 h. Data represent the mean ± SEM of at least five independent experiments. Treatments with TKIs were compared to 0.1% DMSO control with one-way ANOVA followed by Dunnett’s post-test. *p < 0.05 *versus* 0.1% DMSO control.

Superoxide production in mitochondria can be stimulated by toxicants inhibiting complex I (Brand, 2010; Brand, 2016). Accordingly, we determined mitochondrial superoxide accumulation in C2C12 myotubes and in human RD cells exposed to imatinib and dasatinib for 24 h. Mitochondrial superoxide started to increase significantly at 50 µM for imatinib in both cell models (**Figures 4A, B**). Dasatinib increased superoxide accumulation starting at 1 µM in myotubes (**Figure 4C**), but already at 0.2 µM in human RD cells (**Figure 4D**). Erlotinib did not increase mitochondrial superoxide accumulation in myotubes and RD cells, confirming that this specific TKI is not a mitochondrial toxicant up to 20 µM (**Supplementary Figure 1**).

**Figure 5:**
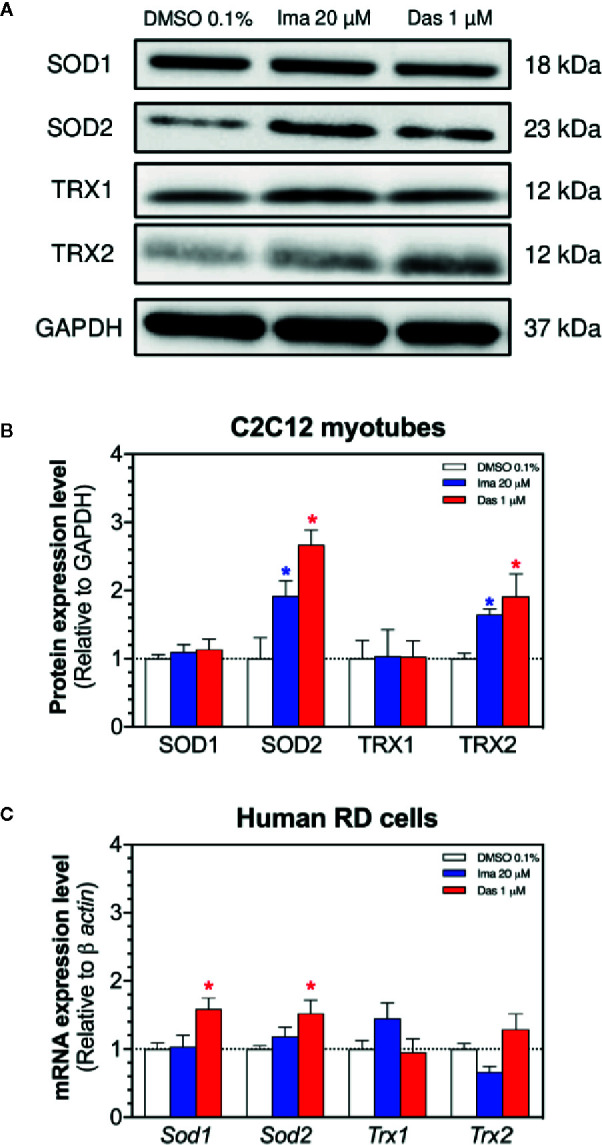
Antioxidative defenses in C2C12 myotubes and human RD cells. **(A)** Western blots showing the expression levels of SOD1, SOD2, TRX1, TRX2, and GAPDH in C2C12 myotubes. **(B)** The graph shows the quantification of protein expression normalized against GAPDH. **(C)** The mRNA expression of genes involved in the antioxidative defense in RD cells. Data represent the mean ± SEM of three independent experiments. Treatments with TKIs were compared to 0.1% DMSO control with one-way ANOVA followed by Dunnett’s post-test. *p < 0.05 *versus* 0.1% DMSO control. Ima, imatinib; Das, dasatinib; SOD, superoxide dismutase; TRX, thioredoxin.

The antioxidative defense system can be induced by mitochondrial accumulation of superoxide. Located exclusively in the mitochondrial matrix, SOD2 degrades mitochondrial superoxide and can be upregulated with the accumulation of mitochondrial superoxide (Fukai and Ushio-Fukai, 2011). Indeed, treatment with imatinib (20 µM) and dasatinib (1 µM) significantly increased the SOD2 protein expression in C2C12 myotubes (**Figures 5A, B**). Protein expression of SOD1 was not affected by treatment with any TKI in myotubes (**Figures 5A, B**). In human RD cells, the exposure to dasatinib increased the mRNA expression of *Sod1* and *Sod2* (**Figure 5C**). However, the mRNA expression of *Sod1* and *Sod2* was not affected by treatment with imatinib (**Figure 5C**).

Thioredoxins play also an important role in ROS (Reactive Oxygen Species) scavenging and participate in redox-regulatory processes of cells (Hanschmann et al., 2013). We measured the protein expression of TRX1 (cytosolic) and TRX2 (mitochondrial) in myotubes exposed for 24 h to imatinib and dasatinib (**Figures 5A, B**). Both TKIs caused an increase in the protein expression of TRX2 (**Figures 5A, B**), which can also be interpreted as an antioxidative defense reaction. Protein expression of TRX1 was not affected by treatment with any TKI (**Figures 5A, B**). In human RD cells, the mRNA expression of *Trx1* and *Trx2* was not affected by treatment with both TKIs (**Figure 5C**).

### Mitochondrial DNA Copy Number and Mitochondrial Proliferation in C2C12 Myotubes and in Human RD Cells

**Figure 6:**
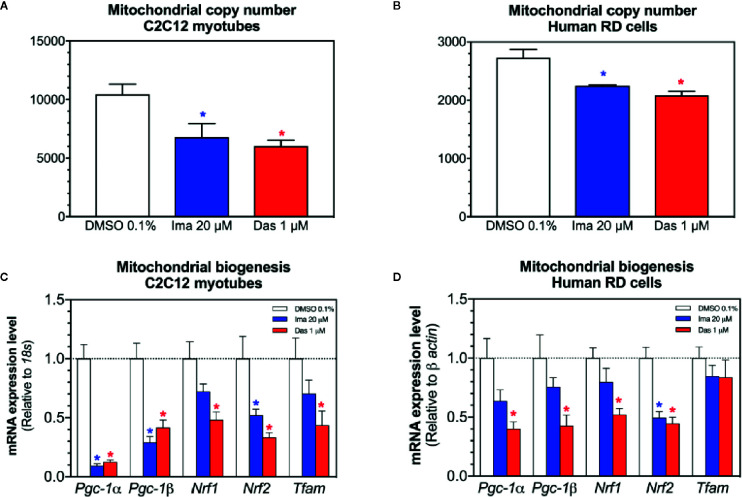
Mitochondrial DNA copy number and markers of mitochondrial biogenesis in C2C12 myotubes and human RD cells exposed to imatinib and dasatinib for 24 h. **(A, B)**. Mitochondrial DNA copy number in C2C12 myotubes and RD cells, respectively. **(C, D)** The mRNA expression of *Pgc-1α*, *Pgc-1β*, *Nrf1*, *Nrf2*, and *Tfam* involved in mitochondrial biogenesis in C2C12 myotubes and RD cells, respectively. Data represent the mean ± SEM of at least three independent experiments. Treatments with TKIs were compared to 0.1% DMSO control with one-way ANOVA followed by Dunnett’s post-test. *p < 0.05 *versus* 0.1% DMSO control. Ima, imatinib; Das, dasatinib; *Pgc-1*, peroxisome proliferator-activated receptor gamma coactivator 1; *Nrf*, nuclear respiratory factor; *Tfam*, mitochondrial transcription factor A.

Mitochondrial toxicants impairing the ETC and causing superoxide accumulation can damage mitochondrial DNA and affect mitochondrial proliferation (Meyer et al., 2013; Singh et al., 2019). Therefore, we measured the mitochondrial DNA copy number and markers of mitochondrial proliferation. Both imatinib and dasatinib decreased the mitochondrial copy number in C2C12 myotubes and RD cells (**Figures 6A, B**, respectively). Since along with oxidative damage, mitochondrial DNA could also be reduced due to impairment of mitochondrial proliferation. For this reason, the mRNA expression of genes involved in mitochondrial biogenesis was measured in C2C12 myotubes and RD cells. The mRNA expression of the peroxisome proliferator-activated receptor gamma coactivator *α* (*Pgc-1α*), *Pgc-1β*, the nuclear respiratory factor 1 (*Nrf1*), *Nrf2*, and the mitochondrial transcription factor A (*Tfam*) were decreased by imatinib and dasatinib in C2C12 myotubes, suggesting that both TKIs impaired mitochondrial proliferation (**Figure 6C**). In human RD cells, dasatinib reduced significantly the mRNA expression of *Pgc-1α, Pgc-1β, Nrf1, and Nrf2* (**Figure 6D**). Moreover, imatinib decreased significantly the *Nrf2* mRNA expression and reduced the mRNA expression of *Pgc-1α*, *Pgc-1β*, and *Nrf1*, but without reaching statistical significance (**Figure 6D**).

### Cell Death and Atrophy in C2C12 Myotubes and in Human RD Cells

**Figure 7:**
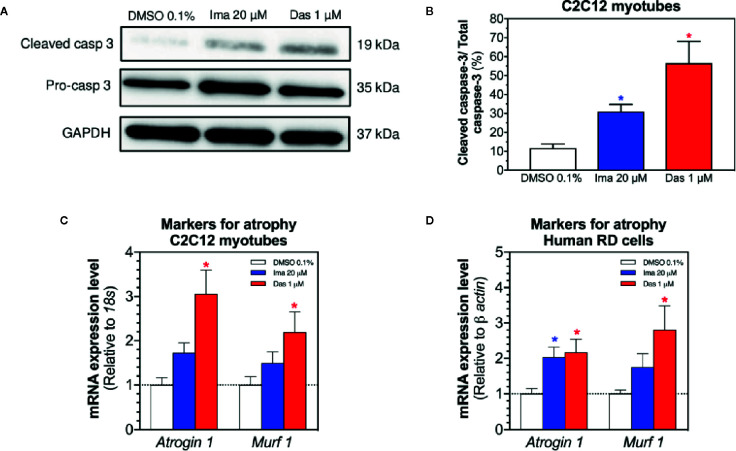
Markers of apoptosis in C2C12 myotubes and of atrophy in myotubes and RD cells exposed to imatinib and dasatinib for 24 h. **(A)** Representative blots of cleaved caspase 3 by western blotting in C2C12 myotubes. **(B)** Quantification of cleaved caspase 3 to the total caspase in C2C12 myotubes. **(C, D)** *Atrogin-1* and *Murf-1* mRNA expression levels as markers of atrophy in C2C12 myotubes and RD cells, respectively. Data represent the mean ± SEM of three independent experiments. Treatments with TKIs were compared to 0.1% DMSO control with one-way ANOVA followed by Dunnett’s post-test. *p < 0.05 *versus* 0.1% DMSO control. Ima, imatinib; Das, dasatinib; *Murf 1*, muscle RING-finger protein-1.

Mitochondrial dysfunction and the presence of oxidative stress can be related with cell death by apoptosis and with atrophy (Green and Reed, 1998; Powers et al., 2012). First, we evaluated the cleavage of caspase 3 in C2C12 myotubes exposed to imatinib and dasatinib for 24 h. Imatinib (20 µM) and dasatinib (1 µM) significantly increased the cleavage of caspase 3 (**Figures 7A, B**). Then, the mRNA expression of genes involved in muscle atrophy was also investigated in C2C12 myotubes and human RD cells. The mRNA expression of Atrogin-1 and muscle RING-finger protein-1 (*Murf-1*) was increased significantly in myotubes and in RD cells exposed to dasatinib (**Figures 7C, D**, respectively). Moreover, imatinib slightly increased the mRNA expression of *Atrogin-1* and *Murf-1* in myotubes and in human RD cells (**Figures 7C, D**, respectively).

## Discussion

In our study, we investigated the mechanisms of myotoxicity associated with three investigated TKIs, which have been described to trigger myopathy in patients. C2C12 myoblasts, myotubes, and human RD cells exposed to imatinib and dasatinib showed a more pronounced decrease in the cellular ATP content compared to membrane damage, a pattern suggesting mitochondrial toxicity. Imatinib and dasatinib reduced the mitochondrial membrane potential and reduced the activity of complex I of the ETC, leading to mitochondrial oxidative stress in C2C12 myotubes and human RD cells. As consequence, the cleavage of caspase 3 and markers of atrophy were increased by imatinib and dasatinib. In contrast, erlotinib was not cytotoxic and did not affect mitochondrial superoxide accumulation in the concentration range investigated.

The effect of imatinib on C2C12 myotubes has already been reported (Damaraju et al., 2018). Indeed, Damaraju et al. showed that exposure to imatinib decreased the ATP content in C2C12 myotubes, which was confirmed in the current study. Our study showed for the first time that the ATP depletion was observed also in human RD cells exposed to imatinib. To the best of our knowledge, the toxicity of dasatinib on C2C12 myoblasts, myotubes, and human RD cells has so far not been described. In the current study, dasatinib was membrane-toxic and decreased the cellular ATP content in C2C12 myoblasts, myotubes, and RD cells. Interestingly, the toxicity of dasatinib in C2C12 myoblasts occurred at much lower concentrations than in the corresponding myotubes. C2C12 myoblasts originally derive from the adult C3H mouse leg muscle and represent an immortalized cell line with similarities to quiescent satellite cells in myofibers (Yaffe and Saxel, 1977). Upon serum removal, C2C12 myoblasts differentiate to multinucleated myotubes that are precursors of mature myofibers (Berendse et al., 2003). During differentiation from myoblasts to myotubes, the expression of myogenin and myosin heavy chain (MHC) increases. Myotubes contain sarcomeres and are able to contract and generate force (McMahon et al., 1994). After the differentiation, the increased expression of muscle-specific genes in myotubes could be responsible for the higher resistance to dasatinib. Importantly, our observations in C2C12 myotubes were confirmed in human RD cells, showing that the myotoxicity observed for imatinib and dasatinib is not confined to C2C12 cells but is a more general finding.

In comparison to imatinib and dasatinib, erlotinib was not toxic for C2C12 cells and human RD cells up to the highest concentration investigated (20 µM). However, fatigue and muscle weakness have been described in up to 19% of patients in a clinical study, which represents one of the most frequent toxicities associated with this drug (Shepherd et al., 2005). In analogy, we have observed a lack of toxicity by erlotinib also in a previous study, where we investigated the toxicity of different TKIs on hepatocellular carcinoma HepG2 cells (Paech et al., 2017). Erlotinib has very limited water solubility, precluding *in vitro* investigations at higher concentrations. It is possible, however, that higher concentrations can be reached in tissues. Interestingly, a female patient with rhabdomyolysis while being treated with the combination erlotinib and simvastatin has been reported (Moscetti et al., 2011). Possible explanations include additive myotoxicity of both drugs and a pharmacokinetic interaction, since simvastatin and erlotinib are both substrates of CYP3A4 (Hidalgo and Bloedow, 2003).

Previously, we and others have shown that mitochondrial dysfunction plays a pivotal role in TKI-associated myocardial and liver toxicity (Kerkela et al., 2006; Will et al., 2008; Mingard et al., 2018; Bouitbir et al., 2019). In the current study, the cellular ATP content was affected at clearly lower concentrations than membrane toxicity for both imatinib and dasatinib, suggesting mitochondrial toxicity. In support of this assumption, exposure of mouse C2C12 myotubes and of human RD cells to 20 μM imatinib or 1 μM dasatinib for 24 h dissipated the *Δψ*m and reduced the complex I activity of the ETC. Regarding imatinib, our results are not in agreement with those of Damaraju et al., who found that the exposure of C2C12 myotubes to up to 50 μM imatinib for 24 h did not dissipate the *Δψ*m but impaired the activity of complex IV of the ETC (Damaraju et al., 2018). One possible reason for this discrepancy between the results of Damaraju et al. and the present study may be the way to assess mitochondrial respiration. We measured mitochondrial respiration with a high-resolution respirometry system, whereas Damaraju et al. determined the activity for each individual enzyme complex separately with enzyme assays. When inhibited, complexes I and III of the ETC are regarded as a source for mitochondrial superoxide production (Brand, 2010; Brand, 2016). As expected, inhibition of complex I of the ETC induced by imatinib and dasatinib was associated with increased accumulation of mitochondrial superoxide. Mitochondrial superoxide accumulation and inhibition of complex I of the ETC in C2C12 myotubes and RD cells exposed for 24 h started at similar concentrations, supporting the concept that superoxide accumulation is most likely a consequence of the impairment of the ETC. Regarding imatinib, our findings are again not in line with the study of Damaraju et al. in which imatinib did not increase superoxide accumulation in C2C12 myotubes (Damaraju et al., 2018). In the current study, mitochondrial superoxide accumulation for both TKIs was observed in both cell models. Mitochondrial ROS was associated with increased mRNA and/or protein expression of SOD2 in both cell models as well as TRX2 protein expression in C2C12 myotubes, which are both located exclusively in mitochondria where they play major roles in ROS scavenging (Holmgren, 1985; Palma et al., 2020). Impaired activity of complex I as the main cause for enhanced mitochondrial superoxide accumulation and the increase of the mitochondrial antioxidative defense suggested that both imatinib and dasatinib can cause oxidative stress, which was shown directly by the rise in the mitochondrial superoxide content.

When mitochondrial ROS production surpasses the capacity of ROS scavenging, oxidative damage to lipids, proteins and DNA can occur (Carraro and Bernardi, 2016). The observed decrease in mitochondrial DNA copy number in both cell models can be considered as a possible consequence of oxidative damage. An additional consequence of oxidative stress is the formation of the mitochondrial membrane permeability transition pore following mitochondrial swelling, which is then associated with apoptosis and/or necrosis (Antonsson, 2004). In our current work, the cleavage of caspase 3 in myotubes exposed to imatinib and dasatinib was increased, indicating apoptosis. This is in line with the study of Damaraju et al. who showed that imatinib enhanced the cleavage of caspase 3/7 in C2C12 myotubes exposed for 24 h (Damaraju et al., 2018). We have shown previously the relationship between mitochondrial superoxide accumulation and sunitinib-associated toxicity in cardiac H9c2 cells by co-incubation with the mitochondria-specific ROS scavenger mito-TEMPO (Bouitbir et al., 2019). If mitochondrial accumulation of superoxide is the driving force for myotoxicity of imatinib and dasatinib, we could expect similar results by mito-TEMPO regarding the toxicity of these compounds on C2C12 cells.

A last possible consequence of oxidative stress is the increased expression of markers involved in atrophy. Oxidative stress is an initial signal and a key contributor to the development of skeletal muscle proteolysis and atrophy (Powers et al., 2007). We showed that the mRNA expression of *atrogin-1* and *Murf-1* increased in both cell models exposed to imatinib and dasatinib. ROS production can contribute to muscle atrophy by promoting the expression of allosteric regulation of proteases (Powers et al., 2012). The relationship between ROS production and atrophy was shown previously where the exposure of myotubes to hydrogen peroxide has been shown to increase the Forkhead transcription factor FoxO3a signaling and the expression of important muscle-specific E3 ligases such as Atrogin-1 and MURF-1 (McClung et al., 2010).

## Conclusions

Our study demonstrates that imatinib and dasatinib were related to decreased complex I activity of the respiratory chain in mitochondria of C2C12 myotubes and human RD cells treated for 24 h. This decreased activity of the respiratory chain was associated with intracellular ATP depletion, a drop in the *Δψ*m, mitochondrial ROS accumulation leading to apoptosis and atrophy. Mitochondrial dysfunction is a likely mechanism of myotoxicity associated with these TKIs. Studies in experimental animals are needed to confirm the results obtained in the *in vitro* experiments.

## Data Availability Statement

The raw data supporting the conclusions of this article will be made available by the authors, without undue reservation.

## Author Contributions

JB, MP, and TF conducted the experiments with cells, interpreted data, and prepared the figures. JB, NR, and SK helped in designing the study, discussed and helped in the interpretation of the data. JB and SK prepared the final version of the manuscript.

## Funding

The study was supported by a grant from the Swiss National Science foundation to SK (SNF 31003A_156270).

## Conflict of Interest

The authors declare that the research was conducted in the absence of any commercial or financial relationships that could be construed as a potential conflict of interest.
