# Supplementary material for: Imatinib and Dasatinib Provoke Mitochondrial Dysfunction Leading to Oxidative Stress in C2C12 Myotubes and Human RD Cells
Source: Front Pharmacol. 2020 Jul 23;11:1106. doi: 10.3389/fphar.2020.01106 (PMC7390871; doi:10.3389/fphar.2020.01106)
Supplement: Supplementary file 1 [file DataSheet_1.docx]

**Supplemental file**

**Imatinib and dasatinib provoke mitochondrial dysfunction leading to oxidative stress in C2C12 myotubes and human RD cells**

**Jamal Bouitbir^1,2,3*^, Miljenko Valentin Panajatovic^1,2^, Theo Frechard^1,2^, Noëmi Johanna Roos ^1,2^, and Stephan Krähenbühl^1,2,3^**

^1^Division of Clinical Pharmacology & Toxicology, University Hospital of Basel, Switzerland

^2^Department of Biomedicine, University of Basel, Switzerland

^3^Swiss Centre for Applied Human Toxicology (SCAHT), Basel, Switzerland

***Correspondence:**

Jamal Bouitbir, PhD

Clinical Pharmacology & Toxicology

University Hospital

4031 Basel, Switzerland

Phone: +41 61 265 2395

Fax: +41 61 265 5401

E-mail: [jamal.bouitbir@unibas.ch](mailto:jamal.bouitbir@unibas.ch)

**Suppl Table 1**

Quantification of membrane toxicity and ATP depletion by tyrosine kinase inhibitors in C2C12 myoblasts and myotubes. Abbreviations: IC_50_: concentration of toxicant with 50% of maximal effect.

|  | IC_50_ membrane toxicity (μM) | | IC_50_ ATP depletion (μM) | |
| --- | --- | --- | --- | --- |
|  | Myoblasts | Myotubes | Myoblasts | Myotubes |
| Imatinib | > 100 | > 100 | 36.1 | 34.9 |
| Erlotinib | 15.1 | > 20 | > 20 | 11.7 |
| Dasatinib | 0.65 | 5.14 | 0.25 | 2.24 |

**Suppl. Table 2**

Quantification of membrane toxicity and ATP depletion by tyrosine kinase inhibitors in human RD cells. Abbreviations: IC_50_: concentration of toxicant with 50% of maximal effect

|  | IC_50_ membrane toxicity (μM) | IC_50_ ATP depletion (μM) |
| --- | --- | --- |
| Imatinib | 93.8 | 28.1 |
| Erlotinib | > 20 | > 20 |
| Dasatinib | 8.31 | 5.18 |
