# Supplementary figures and images for: Imatinib and Dasatinib Provoke Mitochondrial Dysfunction Leading to Oxidative Stress in C2C12 Myotubes and Human RD Cells
Source: Front Pharmacol. 2020 Jul 23;11:1106. doi: 10.3389/fphar.2020.01106 (PMC7390871; doi:10.3389/fphar.2020.01106)

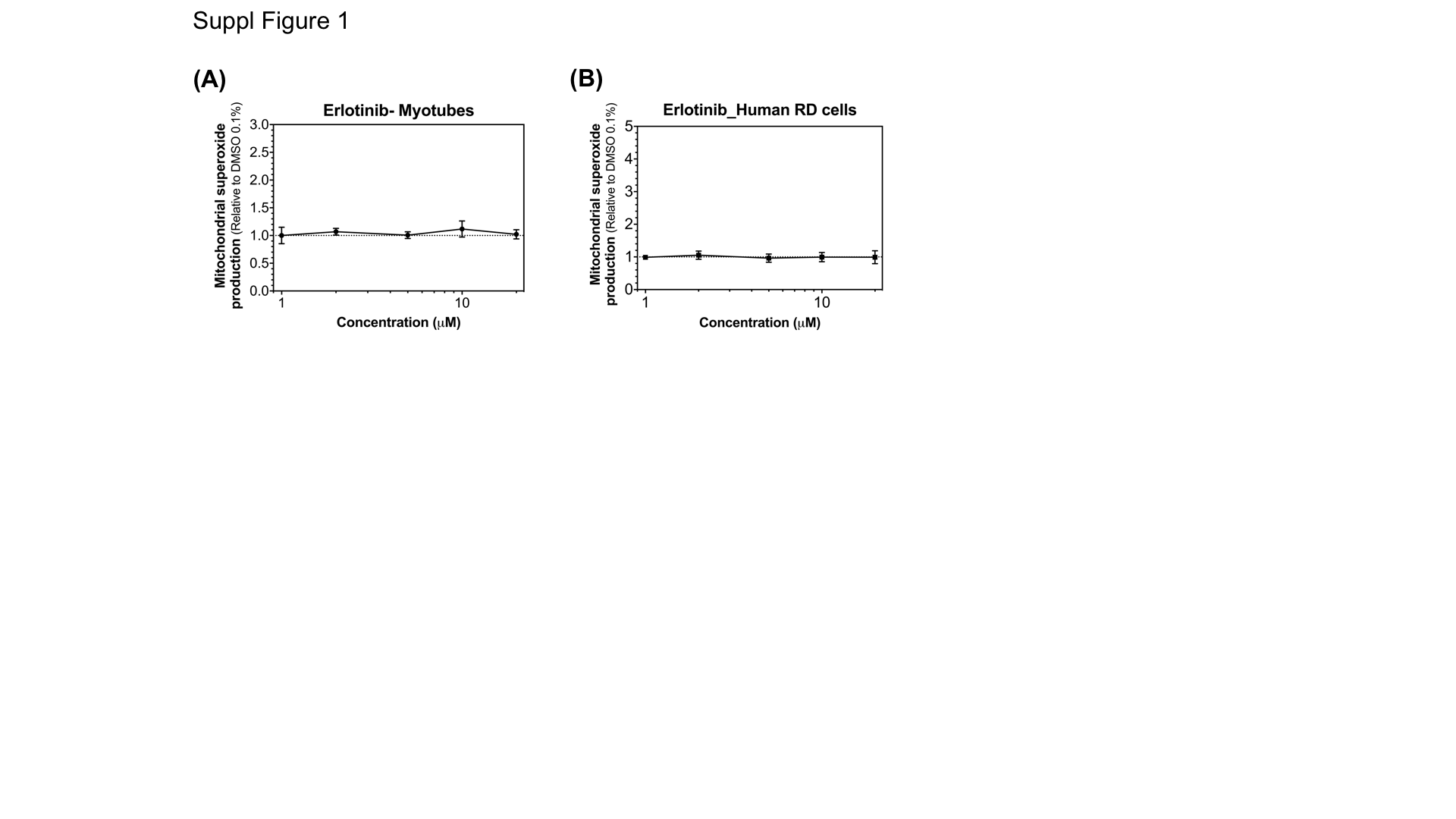

Supplement: Supplementary file 2 [file Image_1.tiff]
